# From pathogenic mechanisms to therapeutic perspectives: a review of gut microbiota and intestinal mucosal immunity in inflammatory bowel disease

**DOI:** 10.3389/fimmu.2025.1704651

**Published:** 2025-11-11

**Authors:** Tianqi Han, Yiwen Zhang, Gang Zheng, Yi Guo

**Affiliations:** The Third Affiliated Clinical Hospital of Changchun University of Chinese Medicine, Changchun, China

**Keywords:** inflammatory bowel diseases, gut microbiota, intestinal mucosal immunity, metabolites, microbiome-based intervention strategies

## Abstract

Inflammatory bowel diseases (IBDs), which comprises Crohn’s disease (CD) and ulcerative colitis (UC), is a multifactorial disorder with an as-yet undetermined etiology, with its global incidence rising rapidly, particularly in developing and Western countries. Although the exact etiology remains unclear, recent research implicates genetic predisposition, environmental factors, gut microbiota, and immune responses in the pathogenesis of IBD. Notably, dysbiosis of the gut microbiota—characterized by a reduction in the abundance and diversity of specific bacterial genera—has been suggested as a potential trigger for the onset of IBD, accompanying with dysregulated intestinal mucosal immunity involving in immune cells and nonimmune cells. Understanding and restoring the imbalanced gut microbiota, as well as identifying key bacterial species involved in IBD, are critical for elucidating disease mechanisms and developing therapeutic strategies. In this review, we explore the role of gut microbiota and intestinal mucosal immunity in the pathogenesis of IBD and offers insights into microbiota-centered therapeutic interventions, including probiotics, fecal microbiota transplantation, and microbial metabolites, that aim to modulate the gut microbiota for the treatment of IBD.

## 1 Introduction

Inflammatory bowel diseases (IBDs), which consists of Crohn’s disease (CD) and ulcerative colitis (UC), are characterized by diarrhea, abdominal pain, bleeding, and extra-intestinal manifestations (1, 2). Specific features contribute to the diagnosis of CD as opposed to UC. These features include small intestinal or upper gastrointestinal involvement, the development of fistulas or abscesses, and transmural inflammation (3, 4). While the classic symptom of UC is rectal bleeding, that is reported by more than 90% of patients (5). Since the year 2000, the global prevalence of IBD has been on the rise in certain industrializing nations (6) as well as among immigrant groups relocating to industrialized countries (7), and it affects up to 1 in 200 individuals in Western countries (4). It is anticipated that the prevalence of IBD will increase by approximately 30% over the next decade in developed countries (8, 9). Moreover, estimates suggest a similar trend in newly industrialized countries (10). The increasing incidence and prevalence of IBD bring a heavy medical burden, mainly represented by drugs (11). However, certain evidence has shown that as many as 40 percent of patients with IBD do not respond to specific drugs. For instance, anti-TNF-α therapy exhibits a primary non-response rate of around 30%. This situation underscores the necessity for a more profound understanding of the pathogenesis of IBD (12, 13).

The etiology of IBD is complex, which is mainly related with genetic susceptibility, environmental triggers, dysregulated immune response, and a dysbiotic gut microbiota (1, 14). Numerous pieces of evidence from epidemiological, genomic, interventional, and *in vitro* studies have illustrated the crucial role of the gut microbiota in the pathogenesis of IBD (12, 15). Factors such as diet (16–18), antibiotics (19, 20), drugs (21), and environment (13, 22, 23) can all cause alterations in the gut microbiota. Multiple lines of evidence have indicated that the gut microbiota composition in patients with IBD differs from that of healthy individuals. This disparity is characterized by a decline in microbial diversity, a reduction in the relative abundance of specific bacterial taxa such as butyrate-producing bacteria, and an increase in opportunistic species, often known as pathobionts (15, 24). The “hygiene hypothesis” has been put forward to account for the rising incidence of IBD in newly industrialized countries. In these regions, reduced childhood exposure to gastrointestinal (GI) pathogens, along with increased use of antibiotic treatments, has given rise to a less tolerogenic gut immune system. As a result, this system becomes more vulnerable to inflammation (25). Furthermore, longitudinal investigations into patients with IBD have demonstrated that the gut microbiota of these patients experiences a transient phase of “dysbiosis” accompanied by metabolic and transcriptional alterations within the gut (26–28). Many studies have showed that fecal microbiota transplantation (FMT) and probiotic therapies are effective in the treatment of mouse models of colitis, while as such are not currently used in clinical practice, due to the variable efficacy (29, 30). This underscores the necessity for a more in-depth comprehension of the particular host signaling pathways that are modulated by the gut microbiome in the setting of IBD.

**Figure 1:**
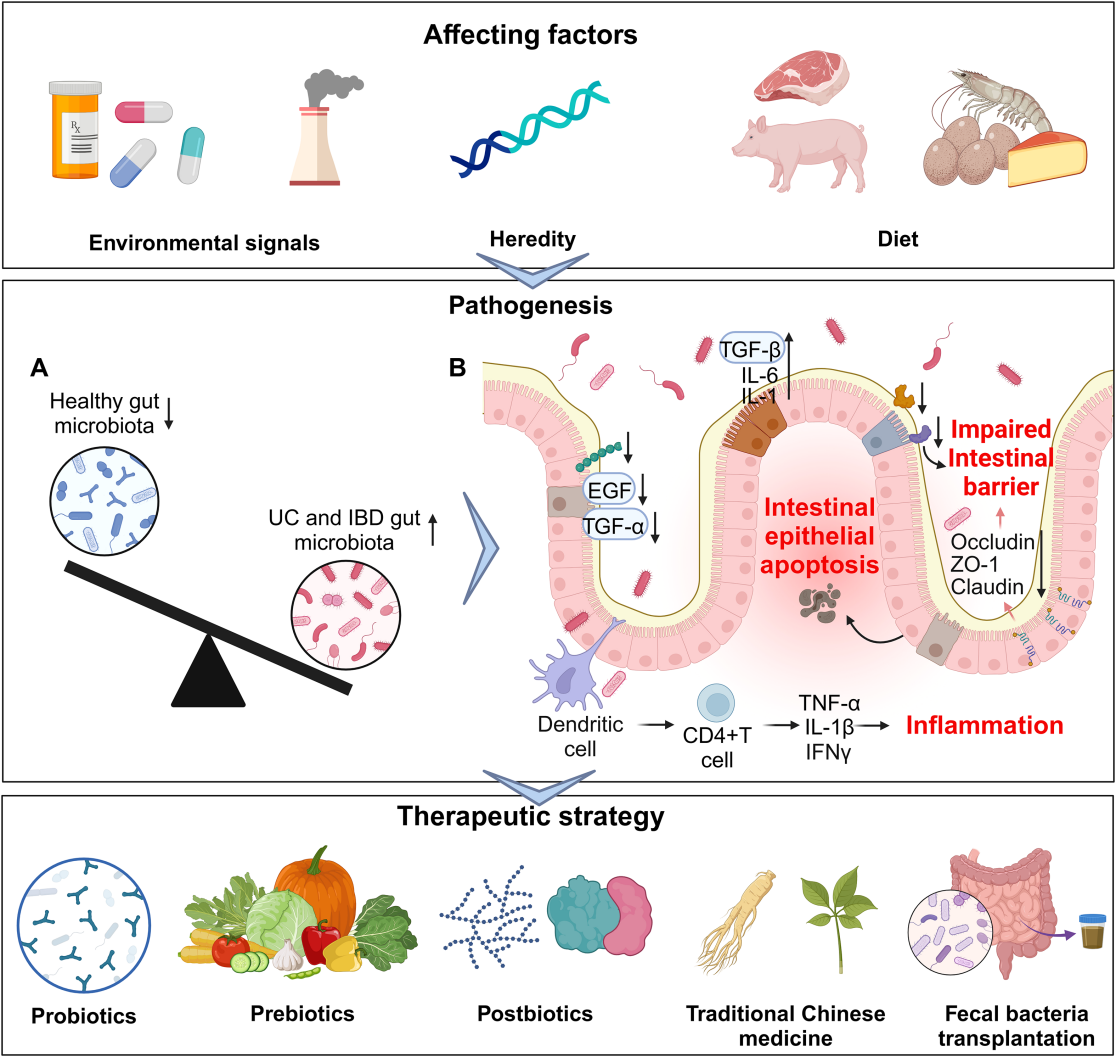
An overview of gut microbiota and related factors in IBD. From affecting factors (upper part), pathogenesis (middle part) to therapeutic strategy (lower part). **(A)** Imbalanced gut microbiota in IBD. **(B)** The influence of the gut microbiota on different cells in IBD.

Over the past few years, advancements in next-generation sequencing and high-throughput technologies, such as metagenomics, metabolomics, and proteomics, have enhanced our capacity to systematically elucidate the specific roles of bacterial strains, metabolites, proteins, and small molecules in the pathogenesis of IBD (31). In this review, our objective is to delineate the current associations between gut dysbiosis and IBD, the host-microbial interactions occurring in both healthy states and the IBD context, as well as the potential clinical applications of microbiota-centered therapeutic strategies for human IBD (**Figure 1**).

## 2 A healthy gut microbiota in the gut

The human body is colonized by ~10^14^ microorganisms that include bacteria, fungi, archaea, protozoa, and viruses, termed the microbiota, and the ratio of bacteria number to human cells is almost 1:1 (32). The GI is the largest microbial niche and is dominated by the bacterial (at least 90%), including phyla such as Bacillota (previously known as Firmicutes), Bacteroidota (previously named Bacteroidetes), Actinobacteriota, Pseudomonadota (formerly Proteobacteria), and Verrucomicrobiota. Among them, the top two phyla, Bacillota and Bacteroidota, account for approximately 90% of the gut microbiota (33, 34). As is widely acknowledged, a central aspect of microbiome research lies in comprehending how diseases affect the composition of the microbiome and vice versa. However, a problematic issue persists: how can we precisely define the terms “healthy gut” and “gut dysbiosis”? Approximately 15 years ago, the concept of “gut health” gradually emerged in the medical literature (35). Typically, from a functional or clinical perspective, gut health was conventionally defined as the absence of any diagnosed digestive diseases or disorders. Nevertheless, this narrow definition may fail to account for the fact that not all issues related to gut health meet the strict criteria for a formal medical diagnosis. A more comprehensive understanding of gut health places great emphasis on the lack of gastrointestinal symptoms as a key indicator. Even though symptoms like bloating, flatulence, irregular bowel movements, and abdominal discomfort may not invariably be linked to a specific disorder, they can still denote a state of suboptimal gut health (34). This definition implicitly underscores the reliance on a well-balanced gut microbiota, as disruptions in microbial balance frequently result in such discomforts.

The human microbiome exhibits an extensive diversity and substantial inter-individual variation. Research findings have indicated that the human gut microbiome can be composed of hundreds to thousands of bacterial species, and significant disparities are observable at higher taxonomic ranks (36). Despite such diversity, certain core microbial species are persistently present in a substantial proportion of the population. This presence implies a common set of functions that are crucial for human health, which is referred to as the core microbiome (37, 38). Hul et al. discussed key characteristics of a healthy gut microbiota, including diversity, composition, functionality, metabolites, strain specificity, gases, pH levels, inflammation markers, and resilience (34). Recently, Wu et al. proposed a guild-based approach that is genome-specific, independent of existing databases, and centered on microbial interactions. This method identifies a core microbiome signature, which can function as a comprehensive health indicator and a potential universal target for promoting health (39).

## 3 Gut microbiota and IBD

Numerous investigations have documented substantial disparities in the composition of gut microbiota between individuals suffering from IBD and healthy controls. These differences are especially prominent regarding microbial diversity and the relative abundance of specific bacterial taxa. In patients with IBD, it is well-established that inflammation and microbial dysbiosis co-occur. However, the question of whether gut dysbiosis serves as a causative factor in the pathogenesis of IBD or merely represents a consequence of the inflammatory process remains an area of active research. Prospective cohort studies designed to tackle this issue pose particular challenges. Nonetheless, a recent study exploited samples obtained from healthy first-degree relatives of CD patients, which were collected via the Crohn’s and Colitis Canada’s Genetic Environmental Microbial (GEM) project, for the purpose of identifying microbial taxa associated with the future onset of CD (40). By applying machine learning techniques, a Microbiome Risk Score was formulated for 70 patients who subsequently developed CD within a cohort of 3,483 participants. This analytical process singled out several pivotal microbial taxa as significant predictors of CD onset. These taxa included *Ruminococcus torques* and *Blautia*, both of which are mucin-degrading bacteria, along with *Colidextribacter*, members of the *Oscillospiraceae* family, and *Roseburia* (40). Furthermore, previous studies have identified functional alterations in the microbiota, including elevated bacterial proteolytic activity, in healthy individuals who subsequently developed UC (41). Ning et al. carried out a comprehensive analysis of nine metagenomic cohorts (with a total of N = 1363 cases) and four metabolomics cohorts (with a total of N = 398 cases) of patients with IBD from various countries or regions by means of cross-cohort integrative analysis. They observed that a substantial reduction in commensal gut microbiota, which plays critical roles in the host’s physiological processes, has been detected in IBD. Several butyrate-producing species (42, 43), namely *Faecalibacterium prausnitzii*, *Roseburia intestinalis*, *Eubacterium hallii*, *Gemmiger formicilis*, *Eubacterium rectale*, and *Ruminococcus bromii*, were found to be significantly diminished in the gut microbiota of patients with IBD (44). Additionally, bacteria engaged in other essential intestinal metabolic processes, such as *Collinsella aerofaciens* (involved in iron metabolism) (45), *Ruminococcus torques* (involved in bile acid metabolism) (46), and *Bifidobacterium longum* (involved in urea cycle metabolism) (47), also exhibit significant reductions. Furthermore, bacteria with antagonistic effects against pro-inflammatory microorganisms, such as *Alistipes putredinis*, are substantially decreased. *Alistipes putredinis* has been demonstrated to exhibit a negative correlation with the colonization of *Candida albicans*. *Candida albicans* is found to be enriched in the guts of patients with IBD and can exacerbate intestinal inflammation via the induction of Th17 cell differentiation (48, 49). Notably, a comprehensive analysis across six separate IBD cohorts revealed that two particular microbial species, *Asaccharobacter celatus* and *Gemmiger formicilis*, were consistently diminished. The first identified and best-characterized IBD pathobiont is adherent-invasive *Escherichia coli* (AIEC). The AIEC strain LF82 uses its adhesin FimH to adhere to intestinal epithelial cells (IEC) expressing the carcinoembryonic antigen-related cell adhesion molecule 6 receptor (50). In addition, LF82 can induce the production of inflammatory cytokines independently of LPS from the intestinal epithelial cells and macrophages through FimH (51). Clardy laboratory showed an inflammatory polysaccharide from the cell surface of *Ruminococcus gnavus* induced expression of the inflammatory cytokine TNF-α from cultured dendritic cells (52). Besides, Liu et al. demonstrated that *Akkermansia muciniphila* regulated colon inflammation through interaction with RORγt+ regulatory T-cell via TLR4 (53). These groundbreaking studies serve as a foundation for future longitudinal investigations aimed at tracking microbiota alterations prior to disease onset in at-risk populations, as well as throughout the natural course of disease progression. Such research has the potential to elucidate critical transition phases and identify key microbial taxa that may play a contributory role in the development of IBD.

**Figure 2:**
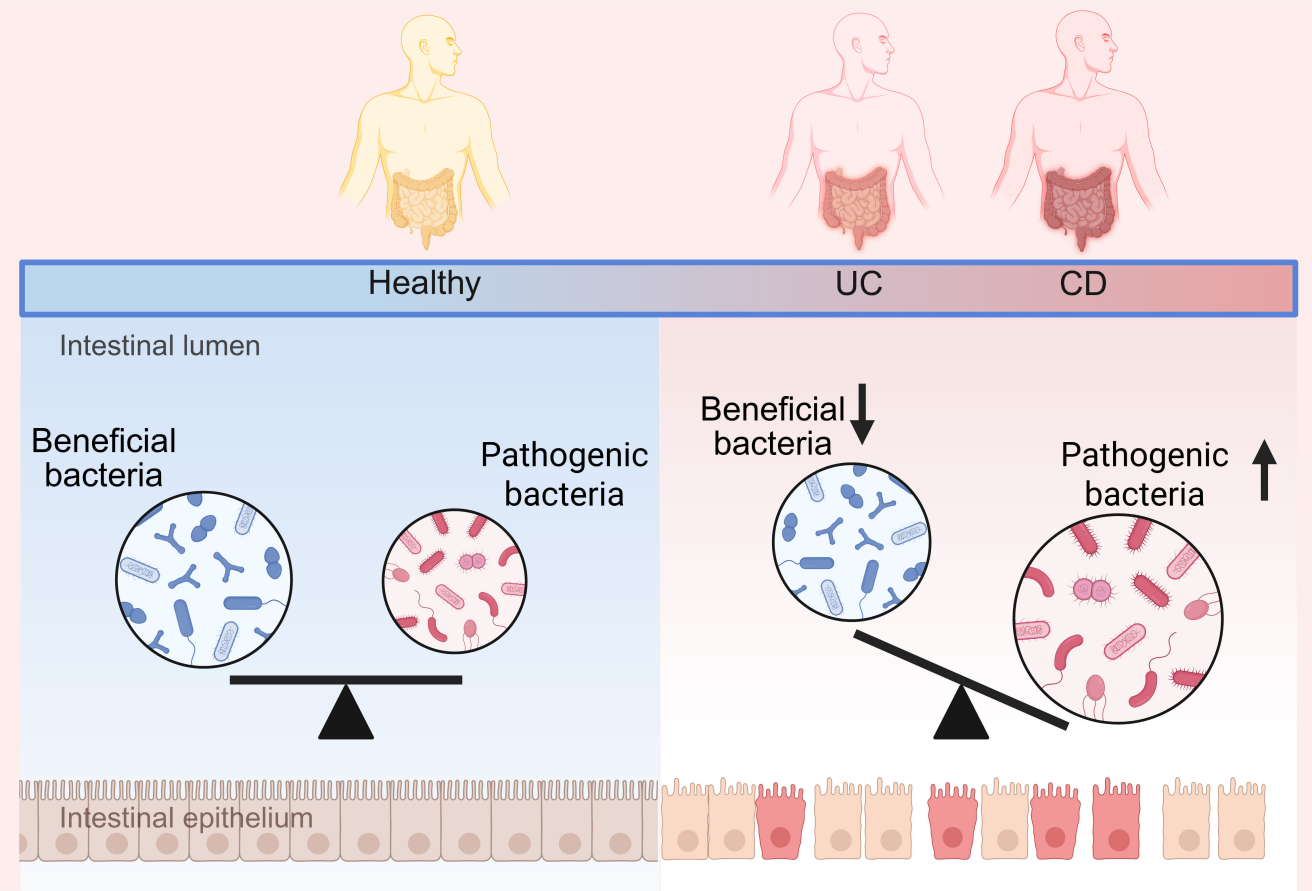
Changes in the gut microbiota in healthy and IBD population.

Sheikh et al. have demonstrated that FMT from healthy individuals and those with defined Crohn’s ileocolitis (CD_L3) into germ-free mice led to a significantly lower engraftment rate of the CD_L3 microbiome in comparison to the healthy control microbiota. Although FMT sourced from CD_L3 patients did not trigger ileitis, it did result in colitis presenting characteristics similar to those of CD. The observed inflammatory response was associated with a sustained increase in the abundance of several bacterial species, namely *Ruminococcus gnavus*, *Erysipelatoclostridium ramosum*, *Faecalimonas umbilicata*, *Blautia hominis*, *Clostridium butyricum*, *Clostridium paraputrificum*, and the unexpected growth of toxigenic *Clostridium difficile*. Notably, the abundance of *Clostridium difficile* was initially below the detection threshold in the inoculated microbial community (54). Gray et al. demonstrated that the engraftment of human-to-mouse FMT displayed greater stochastic variation across individual transplantation events than mouse-adapted FMT. Human-to-mouse FMT induced a population bottleneck, which led to the reassembly of the microbiota composition in a manner specific to the host’s inflammatory environment. In inflamed *IL-10^−/−^* mice, the reassembled microbiota became more aggressive, thereby resulting in more severe colitis upon serial transplantation to other *IL-10^−/−^* mice, in contrast to the distinct microbiota reassembled in non-inflamed WT hosts (55). Recently, a study indicated that DSS treatment did not affect murine colonic microbiota in absence of the host, implying the key role of host-derived components in the affecting colonic microbiota (56). While caution is warranted when extrapolating findings from rodent models to human disease (57), this positive feedback loop between intestinal inflammation and microbial dysbiosis may contribute to the chronic nature of IBD observed in patients. However, microbial dysbiosis likely represents a complex network of bacteria-host interactions, which may vary between individuals depending on their disease stage and baseline microbiota composition (**Figure 2**).

## 4 Innate immune cells in IBD

**Figure 3:**
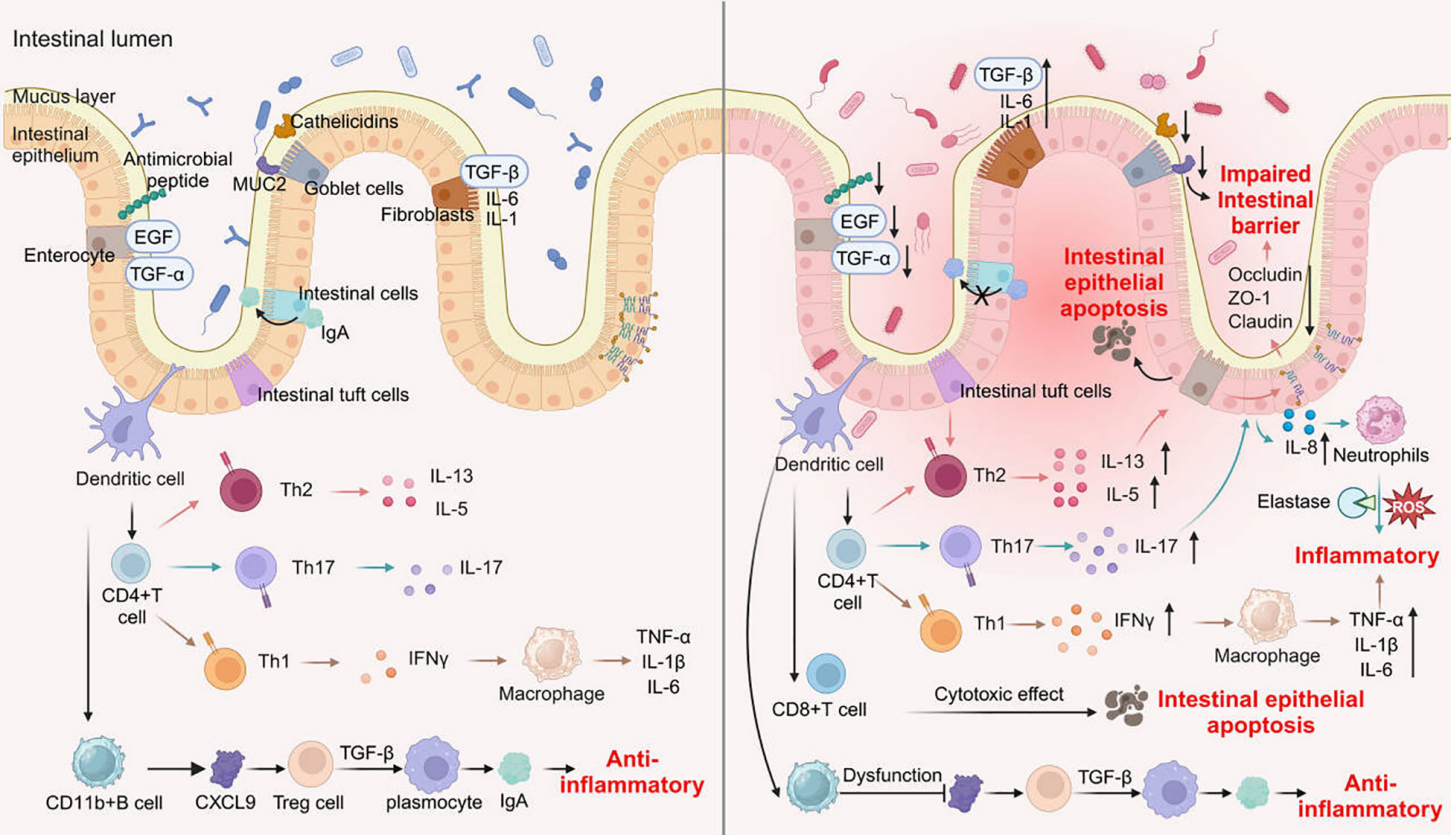
Changes of immune and non-immune cells during intestinal inflammation.

The etiology of IBD is intricate, involving the complex interplay of genetic, environmental, and immune factors. The immune system plays a pivotal role in both the onset and progression of IBD, particularly regarding the roles of innate immune cells and adaptive immune cells in modulating intestinal immune responses (**Figure 3**).

### 4.1 The regulatory role of innate immune cells in IBD

Innate immunity serves as the body’s first line of defense. It is principally constituted by macrophages, dendritic cells, neutrophils, and epithelial cells.

#### 4.1.1 Macrophages

Based on their activation mechanisms, macrophages can be classified into two types: classically activated macrophages and alternatively activated macrophages (designated as M2 type). Classically activated M1 macrophages are triggered by interferon-γ (IFN-γ), TNF-α, and granulocyte-macrophage colony-stimulating factor. Subsequently, they secrete a series of cytokines, including interleukin (IL)-12, IL-23, TNF-α, IL-6, IL-1β, as well as the chemokines CXCL9 and CXCL10. In contrast, M2 macrophages are activated by IL-4, IL-13, and macrophage colony-stimulating factor, and they secrete IL-10 (58).

Macrophages assume a dual function in IBD. On one hand, they initiate the inflammatory process through phagocytosing pathogens and clearing cellular debris. On the other hand, they modulate the immune response by secreting anti-inflammatory factors, such as IL-10 and transforming growth factor-β (TGF-β). In patients with IBD, macrophages frequently display a pro-inflammatory phenotype, over-secreting cytokines including TNF-α, IL-6, and IL-1β, which further aggravates intestinal inflammation. Li et al. reported that macrophage-derived V-domain immunoglobulin domain suppressor of T-cell activation (VISTA) engaged with leucine-rich repeats and immunoglobulin-like domain 1 (LRIG1) and hindered gut epithelial repair in colitis (59). Single-cell RNA sequencing highlights that UC patient-derived macrophages display M1-polarized signatures, with elevated CXCL9 (T-cell chemoattractant) and CD40 (T-cell activation marker), intensifying Th1/Th17-mediated immunity (60). Moreover, macrophages play an essential role in upholding the integrity of the intestinal barrier, since they are capable of promoting the regeneration of epithelial cells via the release of repair factors (61). Fritsch et al. demonstrated that macrophages act as “commensals” that provide metabolic support to promote efficient self-renewal of the colon epithelium in an mTORC1-dependent manner (62). Macrophages are the double-edged sword of IBD, capable of cutting both ways-fueling inflammation or promoting repair. The future of treatment lies in mastering this double-edged sword: harnessing their healing power while sheathing their destructive edge.

#### 4.1.2 Dendritic cells

Dendritic cells represent the most crucial antigen-presenting cells. They identify exogenous pathogens via their surface pattern recognition receptors and subsequently present these antigens to immature T lymphocytes, thereby acting as a vital link between innate and adaptive immunity. Under the condition of normal intestinal homeostasis, dendritic cells remain in a state of immune tolerance.

Dendritic cells are pivotal cells that bridge innate and acquired immunity. These cells possess the ability to capture, process, and present antigens, thereby activating T cells. In patients with IBD, the function of dendritic cells is frequently impaired. This impairment is manifested as a diminished capacity to recognize and present antigens derived from the intestinal microbiota, which in turn gives rise to the dysregulation of the intestinal immune response.

Furthermore, dendritic cells can trigger diverse T cell responses by secreting a variety of cytokines, such as IL-12 and IL-23. This cytokine-mediated induction of T cell responses plays a crucial role in the pathogenesis of IBD. Research findings have indicated that dendritic cells in IBD patients often display a pro-inflammatory phenotype, which may potentially disrupt immune tolerance (61).

#### 4.1.3 Neutrophils

Neutrophils serve as the principal effector cells within intestinal inflammatory responses. In the early phases of IBD, they promptly migrate to the inflammatory site to engage in pathogen clearance. Neutrophils eliminate pathogens through the release of reactive oxygen species (ROS) and multiple enzymes, with elastase being a notable example. Nevertheless, an overabundance of neutrophils can also result in damage to intestinal tissue, thus aggravating chronic inflammation (63). Neutrophils in IBD have revealed unexpected complexities, with heterogeneous populations and dual functions, both deleterious and protective, for the host. Neutrophils modulate the composition and function of the gut microbiota through multiple pathways; conversely, microbial-derived factors-such as metabolites-regulate neutrophil generation and activity via both direct and indirect mechanisms. To advance the development of novel therapeutic strategies for IBD, it is critical to further explore the multifaceted roles of neutrophils in host-microbiota crosstalk, under both homeostatic conditions and inflammatory states.

## 5 Adaptive immune cells in IBD

### 5.1 T cells

T cells constitute a vital component of adaptive immunity, primarily consisting of two major types: CD4+ helper T cells and CD8+ cytotoxic T cells. In the context of IBD, the function and differentiation states of T cells are subject to the influence of multiple factors, which ultimately trigger abnormal immune responses.

#### 5.1.1 CD4+ T cells

CD4+ T cells further differentiate into distinct subgroups, predominantly encompassing Th1, Th2, Th17, and regulatory T cells (Treg). In patients suffering from IBD, the activation levels of Th1 and Th17 cells are frequently elevated. These activated cells secrete pro-inflammatory cytokines, such as IFN-γ and IL-17, which are intimately associated with chronic intestinal inflammation. Th1 cells release copious amounts of TNF and IFN-γ, with IFN-γ modulating the TNF secretion by intestinal macrophages, thereby intensifying the disease severity. Th2 cells play a significant role in UC. Specifically, Th2 cells within the mucosal tissues of UC patients secrete substantial quantities of IL-5 and IL-13. Notably, IL-13 has the capacity to promote the apoptosis of intestinal epithelial cells, thus impairing the integrity of the intestinal mucosal barrier (64). Th17 cells play a crucial immunoregulatory role in IBD. Elevated levels of IL-17, IL-21, and IL-23 have been detected in the mucosa and serum of IBD patients. IL-17A can drive intestinal epithelial cells to secrete IL-8, which in turn triggers the chemotaxis of neutrophils and Th17 cells towards the inflammatory site. Through the signal transducer and activator of transcription 3 (STAT3) pathway, CD4+ T cells facilitate the differentiation and proliferation of Th17 cells. This, in turn, promotes the secretion of IL-17 by Th17 cells, aggravating the inflammatory response. Consequently, inhibiting STAT3 to reduce Th17 cell differentiation can mitigate the inflammatory response in IBD. Conversely, the function of Treg may be suppressed, which results in a diminished tolerance to self-antigens (65).

#### 5.1.2 CD8+ T cells

CD8+ T cells also play a significant role in the pathogenesis of IBD. Principally, they directly induce damage to intestinal epithelial cells via cytotoxic effects, which gives rise to tissue injury. Research findings have demonstrated that the function of CD8+ T cells is activated in patients with IBD, thereby contributing to the aggravation of intestinal inflammation.

### 5.2 B cells

B cells, as a type of multifunctional immune cells, secrete antibodies to mediate humoral immunity. Besides, they are capable of presenting antigens, thus supplying stimulatory signals to T cells and activating the latter to perform immune regulatory functions. B cells are pivotal for antibody production and hold a significant position in adaptive immunity.

In the context of IBD, the malfunction of B cells may also influence disease progression. Studies have indicated that B cell activation is heightened in IBD patients, especially manifested as an increment in the number of plasma cells. These plasma cells can secrete specific antibodies and might be implicated in autoimmune responses.

Wang et al. conducted research demonstrating that, in a DSS-induced colitis model, B cell-deficient mice suffered from more severe disease compared to the normal control groups. Subsequently, when B cells were reintroduced into these B cell-deficient mice prior to DSS administration, the severity of colitis in the mice was alleviated. Moreover, CD11b+ B cells regulate the proliferation of Treg by secreting CXCL9. In turn, Treg cells secrete TGF-β to prompt plasma cells to secrete immunoglobulin A (IgA), which serves to inhibit DSS-induced colitis in mice (66, 67).

Contradictory conclusions regarding the functional roles of immune cells (e.g., macrophages, neutrophils, Th17 cells) in IBD arise from four core causes, including: 1, differences in immune-related genetic polymorphisms and environmental factors across cohorts lead to variations in the responsiveness of immune cells, making it impossible to generalize conclusions across populations. 2, disparities in sample sources (e.g., peripheral blood vs. intestinal lamina propria), detection techniques (e.g., flow cytometry vs. single-cell RNA sequencing), and selection of functional indicators introduce technical biases, amplifying discrepancies in results. 3, In IBD, there is a non-linear interaction between the immune system and gut microbiota. The function of immune cells depends on the microenvironment (e.g., microbial metabolites, intercellular crosstalk), and analysis of single variables easily leads to conflicting conclusions. 4, failure to account for differences in disease stages (active phase vs. remission phase) and intestinal segments results in one-sided conclusions, as the function of the same immune cell varies across different spatial and temporal contexts.

Addressing these contradictions requires targeted optimization of study design, such as stratified analysis of populations, standardization of methods, integration of multi-dimensional data, and incorporation of spatiotemporal factors.

## 6 Non-immune cells in IBD

The non-immune cell population of the intestine constitutes the majority of the cells in the intestinal wall, including two main types: intestinal epithelial cells and stromal cells (68).

### 6.1 Intestinal epithelial cells in IBD

#### 6.1.1 Composition of intestinal epithelial cells

Intestinal epithelial cells are monolayered columnar cells that line the inner surface of the intestine. These cells are composed of absorptive cells, goblet cells, Paneth cells, enteroendocrine cells, intestinal stem cells, along with a small yet diverse subset of other enteroendocrine cell types. Intestinal epithelial cells constitute a crucial site for the absorption and metabolism of nutrients within the body. Additionally, they act as the body’s first line of defense, coming into direct contact with a vast array of intestinal microorganisms (68).

##### 6.1.1.1 Intestinal cells

Intestinal cells are the main cells of the small and large intestines, responsible for the digestion of food and the absorption of nutrients. The tight junctions between intestinal cell membranes serve as a physical barrier to prevent microbial invasion. Intestinal cells produce cytokines to coordinate the response of subcutaneous immune cells. They translocate secretory immunoglobulin A (sIgA) from the basal side of the epithelium to the apical surface of the epithelial cells and then release it into the intestinal lumen. This process plays a vital role in maintaining microbial homeostasis (69).

The tight junctions between intestinal epithelial cells are of critical significance for upholding the integrity of the intestinal barrier. In the context of IBD, the expression of intercellular junctions, including tight junctions and adherens junctions, is diminished, which consequently results in a compromised intestinal barrier function. The downregulation of tight junction proteins, such as claudin, occludin, and zonula occludens-1 (ZO-1), is closely associated with elevated intestinal permeability. This increased permeability enables harmful substances to more readily penetrate the intestinal epithelium, thereby triggering additional inflammatory responses (70).

##### 6.1.1.2 Goblet cells

Goblet cells are an important component of intestinal epithelial cells, primarily responsible for secreting mucus, thereby maintaining the intestinal barrier function and homeostasis. The components of mucus include MUC2, resistin-like molecule β, and trefoil factor. Resistin-like molecule β plays a role in regulating cellular immunity, while trefoil factor promotes the recovery of the epithelium after mucosal injury. Goblet cells can also uptake soluble antigens from the intestinal lumen and transfer them to subepithelial dendritic cells, participating in the secondary immune response (71).

In patients with IBD, the number of goblet cells is significantly reduced, and their mucus secretion ability is diminished, leading to a weakened intestinal barrier and increased susceptibility to pathogens (72).

##### 6.1.1.3 Intestinal tuft cells

Intestinal tuft cells are a morphologically unique type of cell, accounting for about 0.5% of the intestinal epithelium. The marker molecules for tuft cells include Dclk1, Pou2f3, Trpm5, and IL-25. Tuft cells express chemosensory receptors and serve as chemosensory cells in the mucosal epithelium. There is a close interaction between tuft cells and lymphocytes (ILC2s); in cases of intestinal inflammation, tuft cells can recruit T helper 2 cells (Th2) to regulate immune activity in the intestine (73).

### 6.2 Stromal cells in IBD

#### 6.2.1 Composition of interstitial cells

The intestinal interstitial cells mainly include fibroblasts, smooth muscle cells, neurons, and others. These cells regulate physiological processes in the intestine, maintain intestinal barrier function, and participate in immune responses by secreting cytokines and growth factors. For example, fibroblasts play a key role in the repair and remodeling of the intestine, while smooth muscle cells are responsible for intestinal motility.

##### 6.2.1.1 Fibroblasts

Fibroblasts are principally tasked with the synthesis and remodeling of the intestinal extracellular matrix. In the course of enteritis, fibroblast activity is markedly augmented, manifesting as proliferation and the secretion of a diverse array of cytokines. These cytokines exert an influence not only on the local intestinal microenvironment but also on the function of neighboring immune cells. For instance, fibroblasts are capable of secreting TGF-β, IL-1, and IL-6, which fuel inflammatory responses and partake in the intestinal repair process. Moreover, fibroblasts play a vital role in tissue reconstruction following enteritis (74).

In IBD, the activation level of fibroblasts experiences a significant elevation, as evidenced by enhanced proliferative capabilities and augmented cytokine-secretion abilities. Activated fibroblasts generate numerous pro-inflammatory cytokines, including IL-6 and IL-8, which serve to promote local inflammatory responses. Moreover, these cells can potentially modify the intestinal microenvironment through intensified synthesis of the extracellular matrix. This alteration, in turn, may lead to impairment of the intestinal barrier function.

##### 6.2.1.2 Smooth muscle cells

Smooth muscle cells play an important role in the movement and peristalsis of the intestine. In the context of enteritis, the function of smooth muscle cells may change, resulting in a decrease in motility. Studies have shown that enteritis can lead to the remodeling of smooth muscle cells, causing hypertrophy of muscle fibers and functional impairment. These changes may affect the normal peristalsis of the intestine, leading to exacerbation of clinical symptoms such as abdominal pain and diarrhea. In IBD, smooth muscle cells may undergo pathological hyperplasia and functional impairment, resulting in disrupted intestinal motility. The release of inflammatory mediators can directly affect the contraction and relaxation capabilities of smooth muscle cells, thereby impacting the normal function of the intestine (75).

##### 6.2.1.3 Endothelial cells

Intestinal endothelial cells compose the inner lining of blood vessels and are of crucial significance in sustaining intestinal blood flow as well as nutrient supply. In the context of enteritis, both the function and structure of endothelial cells are impacted. Specifically, endothelial cells exhibit enhanced permeability, which gives rise to the leakage of fluids and inflammatory cells. Such an increase in permeability has the potential to aggravate the inflammatory response within the intestine.

Furthermore, during the inflammatory process, endothelial cells express adhesion molecules, such as intercellular adhesion molecule-1 (ICAM-1) and vascular cell adhesion molecule-1 (VCAM-1). These adhesion molecules facilitate the adhesion and migration of leukocytes, thereby further intensifying local inflammation.

Endothelial cells in the intestinal interstitium have also shown alterations in IBD, including increased endothelial cell permeability and disturbed cell alignment. These changes may promote the infiltration of inflammatory cells and exacerbate the inflammatory state of the intestine (76).

## 7 Changes and roles of different intestinal metabolites in colitis

In the pathological progression of colitis, the alterations in gut metabolites have emerged as a key area of research. Gut metabolites not only mirror the functional state of the gut microbiota but also exhibit intricate associations with the host’s health status. Gut metabolites primarily encompass short-chain fatty acids (SCFAs), amino acids, bile salt metabolites, and the like. These metabolites play a significant role in upholding intestinal health, modulating the immune response, and facilitating the repair of intestinal epithelial cells.

### 7.1 Short-chain fatty acids

SCFAs is the main metabolite produced by gut microbial fermentation of dietary fiber, predominantly comprising acetic acid, propionic acid, and butyric acid (77). Research findings suggest that patients with colitis often display reduced levels of SCFAs. This phenomenon is closely associated with a dysbiosis of the gut microbiota (78). SCFAs play a crucial role in mitigating colitis symptoms by suppressing intestinal inflammation, enhancing the integrity of the intestinal barrier, and modulating the immune response. Among various microbial metabolites, butyrate is particularly significant as it participates in multiple signaling pathways within intestinal immune and epithelial cells, thereby aiding in the restoration of compromised colonic barrier function and the maintenance of intestinal homeostasis. Additionally, propionic acid, produced by *Lactobacillus johnsonii*, has been shown to alleviate colitis in murine models by modulating the MAPK pathway to inhibit the polarization of M1 macrophages (79).

### 7.2 Amino acid

Gut microbiota possesses the capability to utilize amino acids for the synthesis of proteins and various metabolites. The metabolism of amino acids within the intestinal microbiome is crucial for the nutritional and physiological well-being of the host (80). In individuals suffering from colitis, amino acid metabolism is frequently disrupted, leading to notable alterations in the concentrations of specific amino acids, such as isoleucine and lysine, which exhibit marked increases in patients with UC (81). Furthermore, plasma levels of tryptophan have been observed to decrease significantly in patients with IBD, and this decrease was negatively correlated with disease activity (82). Tryptophan, as an essential amino acid, can be converted into tryptophan metabolites, such as indole and its derivatives, by intestinal microorganisms, such as *Acinetobacter acidophilus*, *Vibrio cholerae*, *Escherichia coli*, *Pseudomonas aeruginosa* and *Thermosynthophila* (83). Numerous indole derivatives serve as ligands for the aryl hydrocarbon receptor (AhR). The AhR signaling pathway constitutes a crucial element within the immune response at barrier sites. By exerting effects on epithelial cell renewal, barrier integrity, and the immune system, it plays an essential role in maintaining intestinal homeostasis (84). Besides the conventional AhR pathway, indole-3-acetic acid (IAA) mitigates DSS-induced colitis through promoting the production of equol by *Bifidobacterium pseudocolica* (85).

### 7.3 Bile acids

Bile acids (BAs) have emerged as a key class of metabolites associated with the microbiome in patients with IBD. Gut microbiota participates in the biotransformation of BAs through uncoupling, dehydroxylation and recoupling (86). In recent years, metabolomics studies have demonstrated alterations in bile acid metabolism among patients with IBD. Specifically, there is an increase in primary bile acids and a concurrent decrease in secondary bile acids (87). BAs act as potent signaling molecules for the nuclear farnesoid X receptor (FXR). Studies employing animal models have indicated that the activation of FXR contributes to the maintenance of intestinal integrity and serves to prevent DSS-induced colitis. This preventive effect is achieved through the reduction of the production of pro-inflammatory cytokines (15).

## 8 Positive feedback loops in the gut microbiota-metabolite-immune network

While linear causal relationships provide a foundational model for understanding host-microbiome interactions, a growing body of evidence highlights the existence of dynamic, self-reinforcing positive feedback loops between the gut microbiota, their metabolic products, and the host immune system. These loops play a central role in both maintaining immune homeostasis and driving the pathology of chronic diseases, as they serve to amplify initial physiological or pathological signals, leading to the consolidation and persistence of a given state.

### 8.1 Beneficial positive feedback loops in homeostasis

A classic beneficial positive feedback loop aims to consolidate and maintain an anti-inflammatory, tolerant gut environment. The core of this loop often involves immunomodulatory metabolites produced by specific commensal bacteria.

A paradigmatic example is the loop mediated by SCFAs, particularly butyrate. Dietary fiber is fermented by commensals, such as clusters of *Clostridium*, to produce butyrate. Butyrate, in turn, promotes the differentiation and function of colonic Tregs through epigenetic mechanisms, including the inhibition of histone deacetylases (HDACs) (78). The activated Treg cells secrete anti-inflammatory cytokines like IL-10, which effectively suppress the over-activation of effector T cells, thereby maintaining a low-inflammation gut environment. Crucially, this tolerant immune environment provides an ideal niche for butyrate-producing commensals, preventing dysbiosis triggered by excessive inflammation. Thus, a self-reinforcing virtuous cycle is established: Commensals-butyrate-Treg cells-immune tolerance-stable colonization of commensals.

### 8.2 Pathological vicious cycles in disease

Conversely, when an initial event (e.g., infection, dietary disruption, or genetic susceptibility) disrupts homeostasis, positive feedback loops can also pivot towards a malignant direction, accelerating and cementing disease states.

Chronic inflammatory diseases (such as IBD) are often perpetuated by such “vicious cycles”. An initial breach in the mucosal barrier or immune dysregulation may lead to dysbiosis, allowing the overgrowth of pro-inflammatory microbes (e.g., Enterobacteriaceae). These bacteria may produce LPS or other pro-inflammatory substances that activate pattern recognition receptors on host immune cells, triggering signaling pathways like NF-κB and resulting in the release of massive pro-inflammatory cytokines (e.g., TNF-α, IL-6, IL-1β). The resulting highly inflammatory environment further alters gut physiology by increasing oxidative stress, altering mucus composition, and depleting key microbial metabolites like SCFAs. This altered environment is toxic to beneficial commensals but more favorable for inflammation-resistant pathobionts, thereby exacerbating dysbiosis and the severity of inflammation (88, 89).

## 9 Application of gut microbiota therapy in the treatment of colitis

**Figure 4:**
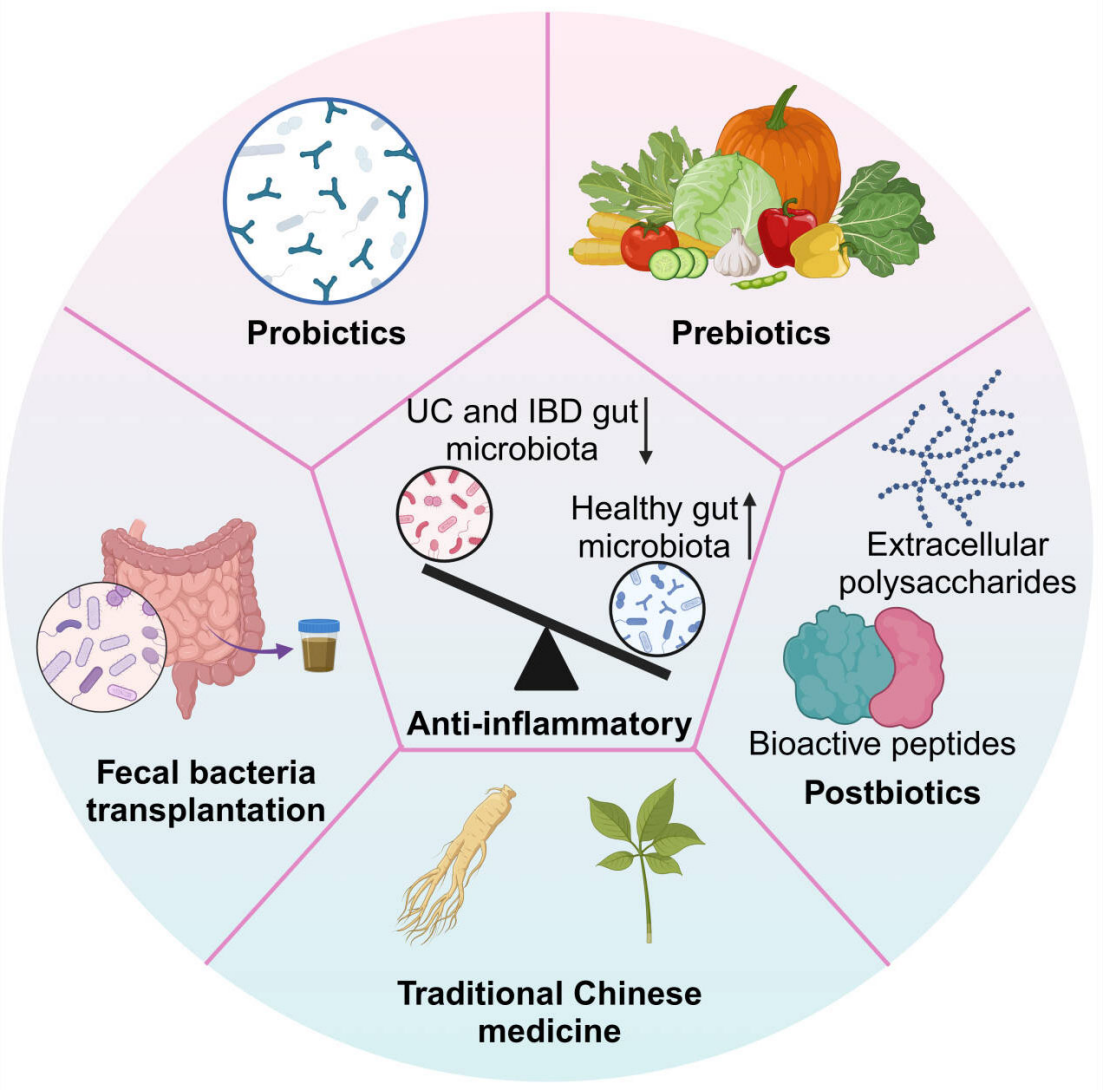
Microbiome-based strategies for IBD.

Conventional treatment modalities for colitis primarily encompass pharmacological interventions, dietary modifications, and surgical procedures. Frequently utilized pharmacological agents include antibiotics, anti-inflammatory medications (such as amino salicylic acid), immunosuppressants, and biologic therapies (90). Prolonged administration of these treatments may result in adverse effects, including the development of drug resistance, heightened susceptibility to infections, and allergic reactions. Furthermore, the efficacy of these pharmacological agents can vary significantly among patients, with some individuals experiencing inadequate responses to existing therapies. Consequently, patients often necessitate long-term medication regimens to manage their symptoms, which not only imposes a financial burden but may also contribute to psychological distress. In recent years, there has been a rapid advancement in research concerning the gut microbiota, leading to the emergence of microbiota-based therapies as a novel approach for the treatment of enteritis. Therapeutic strategies such as probiotics, prebiotics, postbiotics, traditional Chinese medicine, and FMT each possess distinct characteristics and have the potential to enhance intestinal health and mitigate inflammation through various mechanisms (91) (**Figure 4**).

### 9.1 Probiotics

Probiotics are active microorganisms that are beneficial to the host and can help prevent and treat various intestinal diseases by regulating intestinal microbiota, enhancing immune function, and improving intestinal barrier (92). In the treatment of colitis, probiotics can play a role through the following mechanisms: (1) they inhibit the proliferation of pathogenic bacteria by competing for resources and producing antimicrobial substances (93). (2) they stimulate the host’s immune system, thereby increasing resistance to infections and modulating inflammatory responses (94), and (3) they promote the proliferation and repair of intestinal epithelial cells, which strengthens the intestinal barrier and mitigates inflammation (95). Clinical applications of probiotics in colitis treatment have demonstrated efficacy, with research indicating that specific strains, such as *Lactobacillus* and *Bifidobacterium*, can reduce diarrhea incidence and shorten symptom duration in patients with colitis (96, 97). Recently, Guo et al. employed machine learning and bioinformatics techniques to confirm that patients with UC exhibit a decreased prevalence of the *Lactobacillus* genus and an increased level of oxidative stress, and these factors are correlated with the severity of inflammation. Subsequently, they developed a probiotic-based therapeutic approach that synergistically restores the homeostasis of intestinal redox and microbiota. *Lactobacillus casei* (Lac) was induced to form a pericellular film, thereby providing a polysaccharide network for the spatially confined crystallization of ultrasmall yet highly active selenium dots (Se-Lac). Upon oral administration, the selenium dot-embedded pericellular film effectively enhanced the resistance of Lac cells to gastric acid and their intestinal mucoadhesion. At the lesion site, the selenium dots scavenged reactive oxygen species, while Lac modulated the gut microbiota, indicating its promising potential as a treatment for UC (98). However, clinical practice and research evidence reveal multiple unresolved problems and shortcomings that limit their efficacy and widespread application. First, efficacy is limited and highly heterogeneous, failing to meet the needs of generalized treatment. The therapeutic effect of probiotics exhibits significant strain specificity (99). Additionally, individual differences in intestinal microenvironment, genetic background, and disease stages further exacerbate efficacy heterogeneity-what works for one patient may be ineffective for another. Second, safety risks cannot be ignored, especially for vulnerable patient groups. Common adverse effects include gastrointestinal discomfort such as bloating, increased gas, and diarrhea, which are usually transient but may reduce patient compliance (100). Third, lack of standardized protocols plagues the entire chain of probiotic application. There is no unified standard for key parameters such as strain selection, optimal dosage, and treatment duration.

### 9.2 Prebiotics

Prebiotics and postbiotics are two important components that have been widely studied in the field of intestinal health in recent years, and they show a certain application prospect in the treatment of enteritis. Prebiotics mainly include dietary fiber, oligosaccharides (e.g., fructooligosaccharide, inulin), as well as certain resistant starches, serve as substrates for probiotics, thereby promoting their growth and enhancing the intestinal microecological balance (101). Studies have shown that FOS and inulin have been found to have a certain easing effect on intestinal inflammation in some clinical trials (102). Furthermore, the combined administration of prebiotics with probiotics has yielded superior outcomes.

### 9.3 Postbiotics

Postbiotics refer to the metabolites produced during the fermentation of probiotics, including cell wall components, extracellular polysaccharides and bioactive peptides (103). These metabolites have a variety of biological effects on the host, such as antioxidant, anti-inflammatory, immunomodulatory, etc. Epigenome shows potential in the treatment of enteritis. The application of epigenesis in the treatment of enteritis is relatively new, but some studies have shown that they have a positive effect on relieving intestinal inflammation (104, 105). For example, metabolites produced by certain lactic acid bacteria have shown certain efficacy in treating UC, CD, and other diseases (106).

### 9.4 Traditional Chinese medicine

The increasing integration of traditional Chinese medicine (TCM) in therapeutic practices within China has highlighted its effectiveness in managing ulcerative colitis, particularly through the action of specific monomeric components derived from TCM. Comprehensive investigations into these components are likely to yield promising avenues for novel drug development. Chinese herbal medicine has the potential to promote the growth of probiotics and inhibit the proliferation of harmful bacteria by modulating the composition of the intestinal flora. For instance, salidroside enhances the relative abundance of *Lactobacillus* at the genus level and alleviates intestinal inflammation in a manner that is dependent on the gut microbiota (107). Additionally, Chinese herbal medicine can also enhance the intestinal barrier by regulating intestinal functions and promote intestinal repair and regeneration processes. Magnolol and dioscin prevent DSS-induced colitis in mice with enhancing intestinal barrier (108, 109).

The most significant problem in the development of TCM lies in three key aspects: the inability to quantify its efficacy, the lack of unified methodologies and standards, and the ambiguity of markers for TCM quality control. Drawn from practical experience, TCM can only gradually gain entry into the realm of quantitative science by earnestly investigating its mechanism of action through a biological lens and establishing a system for quantifying TCM efficacy-and it is only then that TCM science will embrace a new phase of development. Furthermore, TCM is a personalized medical practice, which enables the customization of treatments to meet individual patients’ needs. However, this inherent heterogeneity creates obstacles to evaluating TCM’s efficacy, ultimately leading to a reduction in the quality of relevant studies. This is an inherent characteristic of TCM: it delivers favorable outcomes for individual patients, yet conducting large-scale, Western-style Randomized Controlled Trials (RCTs) remains challenging. Looking ahead, research on TCM should adopt an approach that allows for minor adjustments tailored to individual patients. Nevertheless, the core TCM formula or herb-acting as the “Emperor” (a key concept in TCM referring to the primary component that targets the root cause of illness)-must be preserved and strictly followed.

### 9.5 Fecal bacteria transplantation

FMT involves the transplantation of fecal matter from a healthy donor into the recipient’s gastrointestinal tract, with the objective of reconstructing the gut microbiome. This therapeutic modality has shown efficacy in the treatment of refractory diarrhea and IBD (110). The primary mechanism underlying FMT is the restoration of intestinal microbiota, which significantly enhances the diversity of probiotics within the gut while simultaneously inhibiting the proliferation of pathogenic bacteria (111). By rebuilding the gut microbiota, FMT improves gut barrier function and reduces gut inflammation. In the treatment of enteritis, FMT is considered to be an effective potential therapy with good tolerance and low incidence of adverse reactions, but its long-term safety necessitates further investigation (112).

However, its current clinical application is still plagued by multi-dimensional limitations and shortcomings, which restrict its clinical promotion and the stability of therapeutic efficacy. First, FMT remission rates vary widely (30%-60% for UC, more unstable for CD), with no unified evaluation standards or stratified data by IBD subtype/stage to identify beneficiaries. Its mechanism (via microbiota reshaping) is unclear, and pre-treatment testing cannot predict efficacy or customize donor microbiota, leading to one-size-fits-all protocols (113, 114). Second, despite donor screening, unknown pathogens/antibiotic resistance genes may transmit infections; post-FMT microbiota shifts can disrupt immune balance, worsening inflammation or triggering autoimmunity (115, 116). In addition, donor screening (eligibility, fecal processing) and administration (route, dosage, frequency) lack unified guidelines, causing inconsistent donor microbiota quality and uncertain efficacy/safety. Donor-recipient matching lacks quantitative indicators, relying on experience (116).

Further clinical studies and randomized controlled trials are crucial to establish their efficacy and safety characteristics more comprehensively. Future research efforts may also explore the combined application of these therapies to optimize therapeutic outcomes. Through ongoing investigations, interventions targeting the gut microbiota are anticipated to play an increasingly central role in the management of colitis (**Table 1**).

**Table 1:** Summary of microbiome-based intervention strategies.

Therapy category	Mechanisms of action	Clinical evidence/Trial status	Limitations
Probiotics	1. Competitive exclusion: competes with pathogens for nutrients and colonization sites. 2. Barrier enhancement: promotes mucus secretion and tight junction protein expression. 3. Immunomodulation: induces anti-inflammatory cytokines (e.g., IL-10), inhibits pro-inflammatory cytokines (e.g., TNF-α). 4. Antimicrobial production: e.g., bacteriocins.	Ulcerative Colitis (UC): Some evidence supports specific strains (e.g., *E. coli* Nissle 1917) for maintaining remission, comparable to mesalazine. VSL<ns/>3 (multi-strain mix) is effective for preventing pouchitis. Crohn's Disease (CD): Overall evidence is weak; efficacy unclear. Status: Widely available as supplements; effects are highly strain-specific (117–119).	1. Efficacy is highly dependent on specific strains and formulations. 2. Limited potency in altering established, complex microbiota. 3. Poor efficacy in severe, active IBD. 4. Lack of standardization; significant variation between products.
Prebiotics	1. Selective stimulation: provides specific nutrients for beneficial bacteria (e.g., Bifidobacteria, Lactobacilli). 2. Indirect benefits: promotes beneficial bacteria to produce SCFAs (e.g., butyrate), which are anti-inflammatory and repair the barrier. 3. Immunomodulation: SCFAs influence dendritic cells and Treg cells.	UC & CD: Inconsistent clinical results. Some studies show improved disease activity indices and increased beneficial bacteria, but many show no significant clinical benefit. Status: Mostly used as dietary supplements (e.g., inulin, FOS); insufficient evidence as a primary therapy (101, 117, 120, 121).	1. Can cause bloating, abdominal pain, and other GI discomfort. 2. Risk of being utilized by harmful bacteria in severe dysbiosis, potentially worsening symptoms. 3. Efficacy depends on the pre-existing population of beneficial bacteria in the host.
Postbiotics	1. Direct action: provides defined bioactive microbial metabolites (e.g., SCFAs, enzymes, surface proteins). 2. Precise immunomodulation: Acts directly on host cells (e.g., epithelial, immune cells) via receptors without requiring live bacteria. 3. High safety: no replication ability; safer for critically ill patients.	Preclinical research: abundant evidence shows anti-inflammatory and barrier-protective effects of butyrate, bacterial polysaccharides, etc. Clinical Status: In early to mid-stage clinical trials; a highly promising emerging field (122–125).	1. Definitions and standardization are still evolving. 2. Optimal active components, dosage, and delivery systems need refinement. 3. Long-term efficacy and safety require more large-scale clinical validation.
Traditional Chinese medicine	1. Multi-target, holistic regulation: comprehensive effects via multiple active components (e.g., polysaccharides, flavonoids, alkaloids). 2. Modulates microbiota: many herbs (e.g., Coptis chinensis, Astragalus, Ginseng) shown to promote beneficial bacteria and inhibit harmful ones. 3. Anti-inflammatory & Repair: directly inhibits inflammatory pathways (e.g., NF-κB) and promotes mucosal repair.	Clinical practice: widely used as adjunct therapy in China and East Asia; numerous clinical observation reports exist. Clinical trials: Many small-scale studies show positive effects, but there is a lack of large-scale, multi-center, randomized double-blind, high-level evidence. Status: modern mechanistic research is emerging (126–128).	1. Complex composition; active ingredients and mechanisms are often unclear. 2. Difficulties in quality control and standardization. 3. Potential risk of herb-drug interactions. 4. Requires rigorous modern clinical validation for global acceptance.
Fecal bacteria transplantation	1. Ecological reset: transfers the complete microbial ecosystem from a healthy donor to the patient's gut, directly correcting dysbiosis. 2. Functional restoration: introduces missing microbes and their functions, restoring normal microbial metabolic networks.	UC: ~30% of patients achieve clinical remission; efficacy is significant but highly variable. Donor screening and patient selection are crucial. CD: Evidence is limited; efficacy less clear than for UC; still under investigation. Status: Considered a promising investigational therapy (111, 129–131).	1. Long-term safety unknown (e.g., potential transmission of undetected pathogens, long-term metabolic disease risk). 2. Unstable efficacy, highly dependent on donor-recipient matching. 3. Lack of standardized protocols (preparation, delivery). 4. Complex regulatory and ethical considerations.

## 10 Conclusions

Recent efforts to clarify microbiome-IBD interactions have advanced understanding of IBD pathophysiology. Both IBD patients and colitis model mice show altered gut microbial profiles, supporting that probiotics, FMT, and improved dietary quality may alleviate IBD by modulating gut microbiota-highlighting daily diet’s critical role in mitigating IBD pathogenesis. Deeper insight into the gut microbiota’s role in IBD is expected to enable more precise disease management.

Key future research directions include: identifying keystone taxa or microbial metabolites regulating IBD-related immune and intestinal barrier function (aided by single-cell sequencing and multi-omics); conducting large-scale longitudinal studies to define microbial signatures for predicting IBD progression or treatment response; exploring synergy between microbiome-based interventions (e.g., probiotics combined with dietary modifications, optimized FMT); and investigating gut-brain/liver axis roles in microbiome-IBD interactions.

Translating microbiome-based strategies to routine IBD care faces challenges: inter-individual microbial heterogeneity limiting “one-size-fits-all” interventions; inconsistent protocols for FMT and variable probiotic products; insufficient safety and long-term efficacy data; and clinical barriers (e.g., limited clinician awareness, poor reimbursement for FMT, patient education gaps). Collaborative efforts across basic science, clinical practice, regulation, and industry to establish evidence-based standards are needed to accelerate translation into effective, accessible IBD treatments.
